# Connexin26 hemichannels with a mutation that causes KID syndrome in humans lack sensitivity to CO_2_

**DOI:** 10.7554/eLife.04249

**Published:** 2014-11-25

**Authors:** Louise Meigh, Naveed Hussain, Daniel K Mulkey, Nicholas Dale

**Affiliations:** 1 School of Life Sciences, University of Warwick, Coventry, United Kingdom; 2 Division of Neonatal Pediatrics, Connecticut Children's Medical Center NICU, University of Connecticut Health Center, Farmington, United States; 3 Department of Physiology and Neurobiology, University of Connecticut, Storrs, United States; The Scripps Research Institute, United States

**Keywords:** respiratory apnea, CO_2_ chemosensing, gap junctions, hemichannels, human, rat

## Abstract

Mutations in connexin26 (Cx26) underlie a range of serious human pathologies. Previously we have shown that Cx26 hemichannels are directly opened by CO_2_ (Meigh et al., 2013). However the effects of human disease-causing mutations on the CO_2_ sensitivity of Cx26 are entirely unknown. Here, we report the first connection between the CO_2_ sensitivity of Cx26 and human pathology, by demonstrating that Cx26 hemichannels with the mutation A88V, linked to Keratitis-Ichthyosis-Deafness syndrome, are both CO_2_ insensitive and associated with disordered breathing in humans.

**DOI:** http://dx.doi.org/10.7554/eLife.04249.001

Connexin26 (Cx26) is one of 21 connexin genes found in humans (Cruciani and Mikalsen, 2006). The canonical function of connexins is to form gap junctions in which two hexameric connexons, or hemichannels, in closely apposed membranes dock together to form an intercellular channel. However connexins can also function as hemichannels, thereby providing large conductance channels, which allow passage of small molecules such as ATP into the extracellular space (Stout et al., 2004; Wang et al., 2013). We have recently shown that Cx26 hemichannels are directly sensitive to CO_2_ (Huckstepp et al., 2010a; Meigh et al., 2013). When CO_2_ binds to Cx26, it carbamylates K125, forms a salt bridge to R104 and opens the hemichannel (Meigh et al., 2013). Cx26 hemichannels are thus a source of CO_2_-gated ATP release (Huckstepp et al., 2010a).

Mutations of Cx26 are the commonest cause of non-syndromic hearing loss (Cohn and Kelley, 1999; Kelley et al., 2000; Xu and Nicholson, 2013). Some of these mutations cause loss of functional protein, while other mutations result in gap junctions and hemichannels with altered properties. However the effect of these mutations on the CO_2_ sensitivity of Cx26 has never been examined. Some missense mutations of Cx26 cause serious pathologies in humans, such as the very rare ectodermal disorder, Keratitis-Ichthyosis-Deafness (KID) syndrome. KID syndrome involves a combination of deafness, visual impairment, and dermatological abnormalities (Caceres-Rios et al., 1996). About 100 cases have been reported in the literature, and of these around 70% are caused by de novo mutations in Cx26, with the remainder being inherited in an autosomal dominant manner or via germ line mosaicism (Sbidian et al., 2010). To date there are nine missense mutations that can cause KID syndrome (Xu and Nicholson, 2013). The severity of the symptoms of KID syndrome depends on the particular mutation in Cx26 (Janecke et al., 2005; Jonard et al., 2008).

The mutation, Cx26^A88V^, is linked to a very severe form of KID syndrome, which is fatal in infancy (Haruna et al., 2010; Koppelhus et al., 2010). In one of the original reports linking Cx26^A88V^ to KID syndrome, the patient required mechanical ventilation (Koppelhus et al., 2010), suggesting a possible effect of the mutation on the neural control of breathing. In KID syndrome caused by a different missense mutation (G45E), which is fatal within the first year of life, there are also reports of breathing problems. One patient required mechanical ventilation immediately after birth (Janecke et al., 2005) and a second died from breathing failure (Sbidian et al., 2010). Nevertheless, without detailed recordings of cardiorespiratory activity, it is not possible to know whether these patients experienced inadequate central respiratory drive. For other mutations linked to KID syndrome there are no reports of abnormal breathing in the literature.

The reason why the A88V and G45E mutations should cause such pervasive and severe pathology remains unclear as subunits of Cx26^A88V^ and Cx26^G45E^ form both functional gap junctions and hemichannels (Gerido et al., 2007; Mhaske et al., 2013). Expression of Cx26^A88V^ in HeLa cells gives rise to enhanced hemichannel-mediated currents (compared to wild type Cx26, Cx26^WT^) at positive transmembrane potentials and in the absence of extracellular Ca^2+^, leading to the suggestion that this mutation represents a gain of function (Mhaske et al., 2013). The G45E mutation, also causes enhanced hemichannel activity in the absence of extracellular Ca^2+^, and increased permeability to Ca^2+^ (Gerido et al., 2007; Sanchez et al., 2010). A gain of function has therefore been suggested as underlying the actions of this mutation too. Although the absence of extracellular Ca^2+^ opens connexin hemichannels, this condition is unlikely to occur in physiological systems. Thus the consequences of the A88V and G45E mutations on physiologically relevant gating of Cx26 remain unclear.

We identified a patient with KID syndrome caused by a heterozygous Cx26 A88V mutation. This patient failed to breathe spontaneously at birth and initially required mechanical ventilation. Later when he started to breathe spontaneously, he continued to demonstrate periods of apnea and bradycardia. A pneumogram performed at a post-menstrual age of 40 weeks showed abnormal persistence of central apnea lasting ≥20 s and accompanied by periods of bradycardia and prolonged oxygen desaturation (Figure 1). This respiratory pattern is abnormal for the age of the infant and is suggestive of blunted chemosensory control of breathing. Given the previously described role of Cx26 in mediating the CO_2_-dependent drive to breathe (Huckstepp et al., 2010b; Wenker et al., 2012), we considered whether the mutation A88V might alter the CO_2_-sensitivity of Cx26.

**Figure 1.**
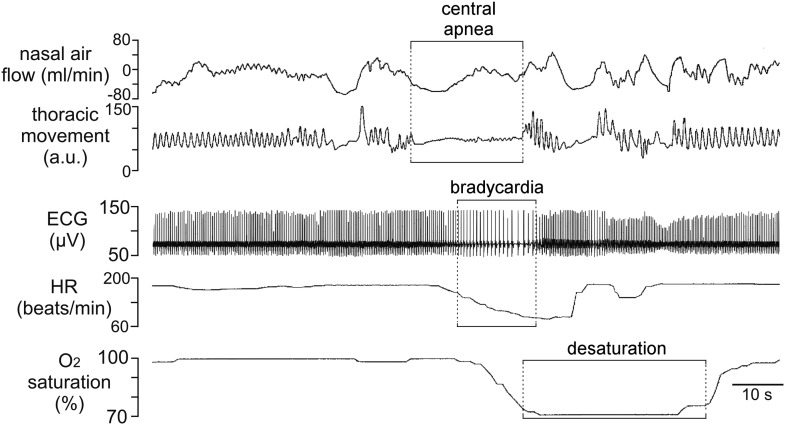
Incidence of central sleep apnea in a patient with Cx26^A88V^. Recording of cardiorespiratory activity during sleep from an infant at a post-menstrual age of 40 weeks diagnosed with KID syndrome. Traces of nasal air flow, thoracic movement, electrocardiogram (ECG), heart rate (HR) and arterial O_2_ saturation show that this patient exhibited a prolonged period during which no effort was made to breathe and this was followed by pronounced bradycardia and arterial O_2_ desaturation, all of which are characteristic of central sleep apnea. Unfortunately, at 2 months of age this patient died from overwhelming sepsis. **DOI:** http://dx.doi.org/10.7554/eLife.04249.002

We introduced the A88V mutation into Cx26 and then tested the CO_2_ sensitivity of Cx26^A88V^ hemichannels expressed in HeLa cells via an established and sensitive dye-loading protocol (Huckstepp et al., 2010a; Meigh et al., 2013). Under conditions of normal extracellular Ca^2+^, HeLa cells expressing wild type Cx26 hemichannels readily load with carboxyfluorescein when exposed to a moderately hypercapnic saline (PCO_2_ 55 mmHg) (Huckstepp et al., 2010a; Meigh et al., 2013). However HeLa cells expressing Cx26^A88V^ showed no such CO_2_-dependent dye loading even when exposed to higher levels of PCO_2_ (70 mmHg, Figure 2). The failure to exhibit CO_2_-dependent dye loading was not due to a lack of functional hemichannels as the positive control of removing extracellular Ca^2+^, which opens all connexin hemichannels, caused robust dye loading (Figure 2). HeLa cells transfected with an empty vector do not show any dye loading in response to a CO_2_ stimulus or removal of extracellular Ca^2+^ (Figure 2—figure supplement 1). Surprisingly therefore, the conservative mutation A88V caused Cx26 hemichannels to lose their sensitivity to CO_2_. As this mutation is far from the residues involved in CO_2_ binding (K125 and R104), the mechanism for the loss of CO_2_ sensitivity is unclear.

**Figure 2.**
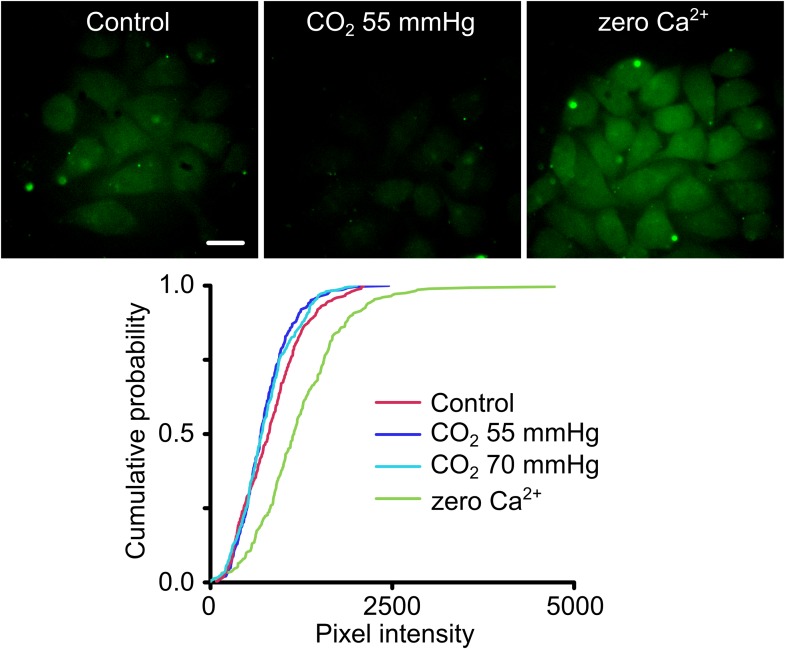
Cx26^A88V^ hemichannels are no longer sensitive to CO_2_. (Top) Images of HeLa cells expressing Cx26^A88V^ under control, hypercapnic and zero Ca^2+^ conditions. The cells were exposed to 200 µM carboxyfluorescein (CBF) for 5 min under each condition before being washed. Some low background loading of CBF is seen under control conditions. In presence of CO_2_ no loading is seen. The positive control of removal of extracellular Ca^2+^ causes robust dye loading demonstrating the presence of functional hemichannels. (Bottom) Cumulative probability distributions of pixel intensity of HeLa cells expressing Cx26^A88V^ under control, hypercapnia (two levels of PCO_2_) and zero Ca^2+^. Only the removal of extracellular Ca^2+^ causes dye loading as shown by the rightward shift of the curve to higher pixel intensities (p = 0.004, Mann Whitney U test compared to control). These distributions show all of the measurements made (minimum 40 cells each from five independent repetitions). **DOI:** http://dx.doi.org/10.7554/eLife.04249.004

**Figure 2—figure supplement 1.**
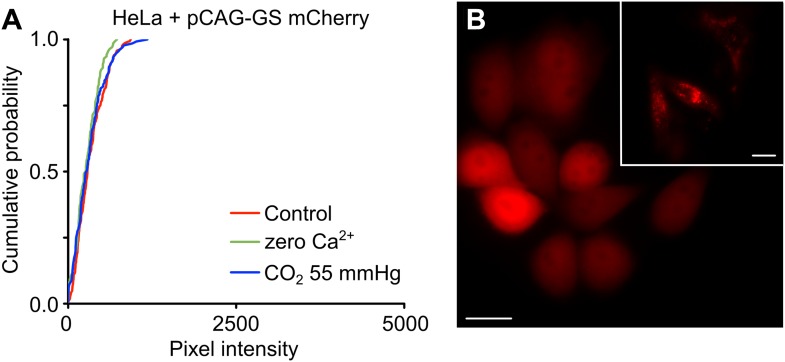
HeLa cells transfected with the empty pCAG-GS mCherry vector show no sensitivity to CO_2_ and do not dye load when exposed to zero Ca^2+^ aCSF. (**A**) Cumulative probability distributions of pixel intensity for HeLa cells transfected with pCAG-GS mCherry under control, hypercapnia and zero Ca^2+^ conditions. The cells were exposed to 200 µM CBF for 5 min under each condition before being washed. The graphs show all of the measurements from 4 independent repetitions for each condition. (**B**) When transfected with pCAG-GS mCherry, the HeLa cells exhibit diffuse red fluorescence from expression of the mCherry. This contrasts with the punctate fluorescence seen flowing transfection with pCAG-GS Cx26-mCherry (inset). Scale bars 20 µm. **DOI:** http://dx.doi.org/10.7554/eLife.04249.003

As the missense mutations which underlie KID syndrome act in a dominant manner (Jonard et al., 2008; Xu and Nicholson, 2013), we tested whether the expression of Cx26^A88V^ subunits might have a dominant negative action on the CO_2_ sensitivity of Cx26^WT^. We transfected HeLa cells that stably expressed Cx26^WT^ with the Cx26^A88V^ subunit and documented their sensitivity to CO_2_ following transfection. 4 days after transfection with Cx26^A88V^, the HeLa cells still exhibited sensitivity to CO_2_ (Figure 3A), but this was reduced compared to the Cx26^WT^ HeLa cells that had not been transfected with Cx26^A88V^ (Figure 3B). 5 and 6 days after transfection, the HeLa cells showed no sensitivity to CO_2_ (Figure 3A). Nevertheless functional hemichannels were still present as the removal of extracellular Ca^2+^ caused dye loading (Figure 3A). The loss of CO_2_ sensitivity was not simply a consequence of days in culture, as Cx26^WT^ HeLa cells that had not been transfected with Cx26^A88V^ retained their sensitivity to CO_2_ over the whole period examined (Figure 3B). We therefore conclude that Cx26^A88V^ subunits are able to act in a dominant negative manner to cause loss of CO_2_ sensitivity from wild type Cx26 hemichannels.

**Figure 3.**
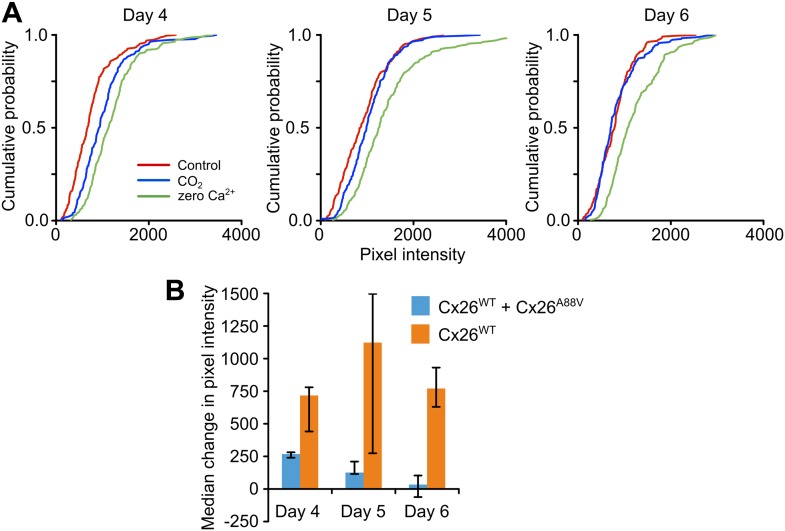
Cx26^A88V^ hemichannels act in a dominant negative manner to remove CO_2_ sensitivity from Cx26^WT^. (**A**) Cumulative probability distributions for CO_2_-dependent dye loading in HeLa cells that stably express Cx26^WT^, which have been transfected with Cx26^A88V^. 4 days after transfection with Cx26^A88V^ the cells still exhibit significant sensitivity to 55 mmHg PCO_2_ stimulus (p = 0.048 CO_2_ compared to control, Mann Whitney U test). 5 and 6 days after transfection the CO_2_ sensitivity of the HeLa cells was abolished. On all 3 days, the positive control of zero Ca^2+^ caused dye loading, demonstrating the presence of functional hemichannels. The graphs show all of the measurements made from 5 independent repetitions of the experiment. (**B**) Comparison of the sensitivity to CO_2_ of HeLa cells stably expressing Cx26^WT^ which have been transfected with Cx26^A88V^ (Cx26^WT^ + Cx26^A88V^, n = 5) with those that have not (Cx26^WT^, n = 7). In the absence of transfection, the Cx26^WT^-expresssing HeLa cells retain sensitivity to CO_2_ on all 3 days. By contrast Cx26^A88V^ causes significantly depressed CO_2_ sensitivity 4 days after transfection (p = 0.001), and loss of sensitivity on days 5 (p = 0.024) and 6 (p = 0.001). Comparisons of Cx26^WT^ with Cx26^WT^ + Cx26^A88V^ via Mann Whitney U test, and False Discovery Rate procedure for multiple comparisons (Curran-Everett, 2000). Error bars upper and lower quartiles. **DOI:** http://dx.doi.org/10.7554/eLife.04249.005

This is the first instance in which a mutation linked to serious human pathologies has been demonstrated to abolish the CO_2_ sensitivity of Cx26. This in turn suggests that Cx26-mediated CO_2_ sensing may be important for human physiology in the range of contexts that are associated with the diverse pathologies linked to this mutation. In the closely related β connexin, connexin30 (Cx30), the mutation A88V connected to Clouston's Syndrome (Bosen et al., 2014), may result in constitutively open Cx30 hemichannels (Essenfelder et al., 2004). However Cx30 is also opened by CO_2_ (Huckstepp et al., 2010a) and the effect of this mutation on the CO_2_ sensitivity of Cx30 has not yet been investigated. There are no reports in the literature of disordered breathing in patients with Clouston's syndrome.

Previous studies suggesting that the A88V mutation gave a gain of function in Cx26, examined hemichannel function in the absence of extracellular Ca^2+^ (Mhaske et al., 2013). As the CO_2_ sensitivity of the mutated hemichannel was not specifically examined in this previous study, it is likely that both sets of findings are correct—an enhancement of macroscopic hemichannel currents (Mhaske et al., 2013), and a loss of CO_2_ sensitivity. However under physiological conditions of normal extracellular Ca^2+^ and in the presence of physiological CO_2_/HCO_3_^−^ buffering, we suggest that A88V should be considered as a loss-of-function mutation that effectively removes the capacity for CO_2_-evoked ATP release via Cx26 hemichannels.

Our report is the first to document altered central respiratory drive in a KID syndrome patient. In rodents, CO_2_-sensitivity of Cx26 contributes to the chemosensory control of breathing (Huckstepp et al., 2010b; Wenker et al., 2012). Although we do not know if the loss of CO_2_ sensitivity in Cx26 contributed to the aberrant respiratory drive exhibited by this patient, these results are consistent with this possibility, and represent the first evidence to suggest that Cx26 hemichannels are a requisite component of the drive to breathe in humans. Overall the ability of physiological levels of PCO_2_ to permit ATP release via Cx26 hemichannels may be important in the epidermis, cochlea and brain. Investigation of whether the absence of this mechanism of ATP release in patients with Cx26^A88V^ contributes to the serious pathological abnormalities that they suffer would seem to be warranted.

## Materials and methods

### Case study

The Institutional Review Board of the Connecticut Children's Medical Center considered this under the category of a case report and thus exempt from formal review.

### Mutant connexin production

Puc19 Cx26^A88V^ was produced from wild type Cx26 via the Quikchange protocol using the following primers: forward 5′ TGT CCA CGC CGG TCC TCC TGG TAG C 3′ reverse 5′ GCT ACC AGG AGG ACC GGC GTG GAC A 3′. Cx26^A88V^ was subcloned into a pCAG-GS mCherry vector for mammalian cell transfection. Successful mutation of Cx26 was confirmed by sequencing which also verified that apart from the desired mutation the sequence was identical to the wild type.

### HeLa cell culture

HeLa cells were cultured by standard methods in DMEM, 10% FCS with addition of 3 mM CaCl_2_. For experimentation, cells were plated onto coverslips at a density of 5 × 10^4^ cells per well. Transient transfections were performed using the genejuice protocol.

### Solutions used

#### Control aCSF

124 mM NaCl, 26 mM NaHCO_3_, 1.25 mM NaH_2_PO_4_, 3 mM KCl, 10 mM D-glucose, 1 mM MgSO_4_, 1 mM CaCl_2_.

#### Zero Ca^2+^ aCSF

124 mM NaCl, 26 mM NaHCO_3_, 1.25 mM NaH_2_PO_4_, 3 mM KCl, 10 mM D-glucose, 1 mM MgSO_4_, 1 mM MgCl_2_, 1 mM EGTA.

#### Hypercapnic (55 mmHg CO_2_) aCSF

100 mM NaCl, 50 mM NaHCO_3_, 1.25 mM NaH_2_PO_4_, 3 mM KCl, 10 mM D-glucose, 1 mM MgSO_4_, 1 mM CaCl_2_.

#### Hypercapnic (70 mmHg CO_2_) aCSF

70 mM NaCl, 80 mM NaHCO_3_, 1.25 mM NaH_2_PO_4_, 3 mM KCl, 10 mM D-glucose, 1 mM MgSO_4_, 1 mM CaCl_2_.

Hypercapnic aCSF was saturated with sufficient CO_2_ (the remaining balance being O_2_) to adjust the final pH (pH 7.5) to that of the control aCSF removing any potential effects of changes in extracellular pH.

All other solutions were saturated with 95% O_2_/5% CO_2_.

### Dye loading protocols

Coverslips plated with HeLa cells transiently transfected with Cx26^A88V^ were exposed to Hypercapnic aCSF (55 mmHg or 70 mmHg) containing 200 µM CBF for 10 min. This was followed by control aCSF with 200 µM CBF for 5 min and a 30 min wash with control aCSF to ensure that all dye is removed from the outside of the cells.

A control comparison was used to establish any baseline loading occurring in the absence of a stimulus. HeLa cells expressing Cx26^A88V^ were exposed to 200 µM CBF in control aCSF for 15 min, followed by 30 min of washing.

A zero Ca^2+^ positive control was also performed to ensure functional connexin hemichannels were being expressed. Cx26^A88V^ expressing HeLa cells were exposed to 200 µM CBF in zero Ca^2+^ aCSF for 10 min. This was followed by control aCSF with 200 µM CBF for 5 min and 30 min of washing with aCSF.

### Imaging and analysis

For each condition cells were imaged by epifluorescence (Scientifica Slice Scope, Cairn Research OptoLED illumination, 60× water Olympus immersion objective, NA 1.0, Hamamatsu ImageEM EMCCD camera, Metafluor software). Using ImageJ, the extent of dye loading was measured by drawing a region of interest (ROI) around individual cells and calculating the mean pixel intensity for the ROI. The mean pixel intensity of the background fluorescence was also measured in a representative ROI, and this value was subtracted from the measures obtained from the cells. All of the images displayed in the figures reflect this procedure in that the mean intensity of the pixels in a representative background ROI has been subtracted from every pixel of the image. The analysis of the CO_2_ sensitivity of Cx26^A88V^ was performed as five independent repetitions in which at least 40 cells were measured in each condition, and the mean pixel intensities plotted as cumulative probability distributions.
